# Breastfeeding patterns and risk of childhood acute lymphoblastic leukaemia

**DOI:** 10.1038/sj.bjc.6602706

**Published:** 2005-07-19

**Authors:** M L Kwan, P A Buffler, J L Wiemels, C Metayer, S Selvin, J M Ducore, G Block

**Affiliations:** 1 Division of Public Health Biology and Epidemiology, School of Public Health, University of California, Berkeley, CA 94720, USA; 2 Department of Epidemiology and Biostatistics, School of Medicine, University of California, San Francisco, CA 94143, USA; 3 Department of Pediatrics, Section of Hematology/Oncology, University of California, Davis, Sacramento, CA 95817, USA

**Keywords:** child, leukaemia, acute lymphoblastic leukaemia, breastfeeding, infection

## Abstract

The risk of childhood acute lymphoblastic leukaemia (ALL) was investigated in relation to breastfeeding patterns in the Northern California Childhood Leukaemia Study. Data collected by self-administered and in-person questionnaires from biological mothers of leukaemia cases (age 0–14 years) in the period 1995–2002 were matched to birth certificate controls on date of birth, sex, Hispanic ethnic status, and maternal race. Ever compared to never breastfeeding was not associated with risk of ALL at ages 1–14 years (odds ratio=0.99; 95% CI=0.64–1.55) and ages 2–5 years (OR=1.49; 95% CI=0.83–2.65). Various measures of breastfeeding duration compared to absence of breastfeeding also had no significant effect on risk. Complimentary feeding characteristics such as type of milk/formula used and age started eating solid foods among breastfed children were not associated with ALL risk. This study provides no evidence that breastfeeding affects the occurrence of childhood ALL.

For several decades, an infectious aetiology has been postulated as a cause of leukaemia in children (Heath and Hasterlik, 1963; Greaves, 1988; Kinlen, 1988; Severson *et al*, 1989; Doody *et al*, 1992), together with supporting evidence (Kinlen, 2000; Ma *et al*, 2002; Perrillat *et al*, 2002a, 2002b; Jourdan-Da Silva *et al*, 2004).

One particular hypothesis by Greaves (Greaves, 1999; Greaves and Wiemels, 2003) holds that common B-cell precursor acute lymphoblastic leukaemia (c-ALL) arises as a consequence of a rare, abnormal response to nonspecific, common infections in two genetic events. As a first step, a spontaneous genetic alteration, often a chromosomal translocation, occurs during the expansion of B-cell precursors in the pre- or perinatal period. Subsequently, a second mutational event arises following antigenic challenge early in life. Support for the first step is seen in genetic backtracking of the *TEL-AML1* translocation, a genetic mutation contributing to c-ALL (Wiemels *et al*, 1999; McHale *et al*, 2003).

Within the context of the Greaves' hypothesis, it is postulated that maternal breastfeeding may protect against childhood ALL by modulating the child's immune system early in life to respond effectively during exposure to common infections later in life. (Davis, 1998; Greaves, 1999). To date, a number of studies have explored the relationship between breastfeeding and childhood leukaemia risk (Kwan *et al*, 2004b) with inconsistent results (Davis, 1998), but the overall trend suggests a protective effect (Davis, 1998; Kwan *et al*, 2004b).

Given that breastfeeding is a potentially relevant immunological exposure during a critical developmental period, we examined this in relation to ALL risk in the Northern California Childhood Leukaemia Study (NCCLS). Initiation, duration, and exclusivity of breastfeeding were investigated along with complimentary early feeding characteristics such as age when the child first drank formula or milk, type of formula or milk consumed, and type of solid food eaten.

## MATERIALS AND METHODS

### Study population

The NCCLS is an ongoing, approximately population-based case–control study that began in 1995. This analysis consists of data collected from two phases of the study: Phase one from 19 August 1995 to 30 November 1999 and Phase two from 1 December 1999 to 30 November 2002. Incident childhood leukaemia cases were identified using the *International Classification of Diseases for Oncology* (ICD-O) criteria (ICDO, 2000) with rapid case ascertainment procedures from seven (Phase one) and expanded to nine (Phase two) pediatric hospitals in the Northern and Central California study region. During Phase one, the study area consisted of 17 counties in Northern California, and then during Phase two, the area was extended to include 18 counties in Central California. Comparison with the statewide California Cancer Registry for 2000 showed that 95 and 76% of eligible cases among residents in the five counties San Francisco-Oakland Metropolitan Statistical Area and in the other 30 counties of the study area, respectively, were identified by the NCCLS protocol. The evaluation of case ascertainment for all 35 counties is currently underway. Cases were eligible if they were under 15 years of age, had no previous history of any malignancy, lived within the study region, and their parents spoke either English or Spanish. The study was approved by the University of California Committee for the Protection of Human Subjects, the California Health and Human Services Agency Committee for the Protection of Human Subjects, and the institutional review boards of the participating hospitals. Written informed consent was obtained from the parents of all participating study subjects.

After each case was identified, a control subject was randomly selected from birth certificates supplied by the California Birth Registry. Birth certificates were matched 1 : 1 (Phase one) or 1 : 2 (Phase two) to the case on date of birth, sex, Hispanic ethnic status (a child is considered Hispanic if either parent is Hispanic), maternal race, and maternal county of residence at birth (in Phase one) and maternal residence in 35 counties of the study area (in Phase 2). For cases not born in California (7%), controls were selected from the case county of residence at diagnosis. Inclusion of cases born out of state was demonstrated to not modify study results in earlier analyses (Jensen *et al*, 2004; Kwan *et al*, 2004a).

As of 1 December 2002, 283 case–control pairs and 100 case–control triplets were available for analysis. Since the immunophenotype and likely the etiology of infant leukaemia differs from the other childhood leukaemias (Ross, 1998, 2000), 19 cases and their respective controls who were diagnosed during the first year of life were excluded, leaving 266 pairs and 98 triplets for analysis. Of these, 222 pairs and 89 triplets were of ALL subtype, and the remaining 44 pairs and eight triplets were of acute myeloblastic leukaemia (AML) subtype and one triplet was of juvenile myelomonocytic leukaemia (JMML) subtype.

**Figure 1:**
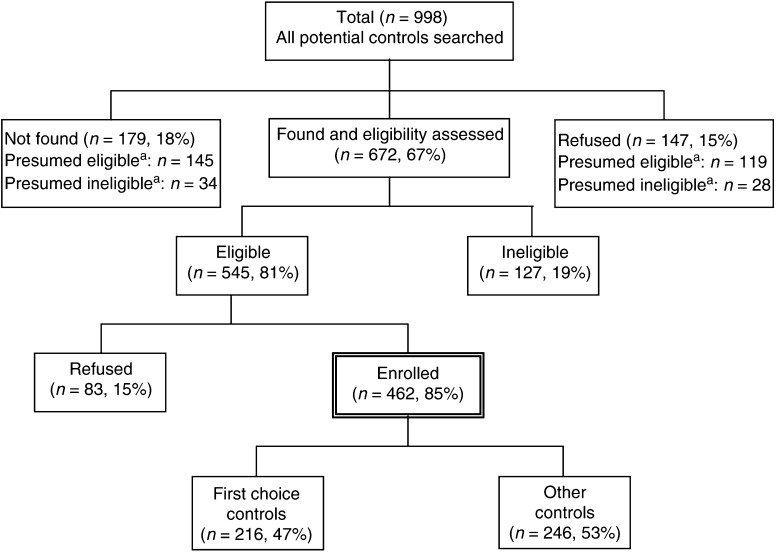
Control selection strategy in the NCCLS for the breastfeeding patterns analysis (19 August 1995 to 30 November 2002). ^a^Assuming the same percentage of eligible as that in potential controls who were found and whose eligibilities were assessed.

The overall case participation rate was 86%, while the overall control participation rate (number of eligible participating controls divided by the total number of eligible controls) was 85%. Including all potential controls (eligible, not located, and refused) in the calculation yielded a participation rate of 57%. Reasons for nonparticipation of the eligible and presumed eligible controls included refusals (25%) and not located (18%). Details of control participation in this analysis are provided in Figure 1. Further details of NCCLS control recruitment is given elsewhere (Ma *et al*, 2004).

### Data collection and management

Information regarding breastfeeding and complimentary feeding characteristics was collected by an in-home interview and a self-administered questionnaire, respectively. Most often, the biological mother provided the information on both instruments (95%). Respondents were asked if they ever breastfed their child for at least 1 day (ever/never) and for how long (in months, weeks, or days). Specific feeding characteristics of interest were the age the child started drinking milk or formula, the type of milk or formula consumed at or before 6 months and after 6 months of age, the age child started eating solid foods, and the type of solid food consumed.

Breastfeeding was analysed as a binary (ever/never) variable. Duration of breastfeeding was analysed as reported (continuous) in months and as categories of none, less than, or equal to 3 months, 4–6 months, 7–12 months, and greater than or equal to 13 months. Exclusive breastfeeding (in relation to consuming other milk or formula) was derived from duration of breastfeeding (continuous) and the age when the child was first introduced to formula or milk (categorical) and was categorised as formula only, breast milk only for less than or equal to 3 months, 4–6 months, 7–12 months, and greater than or equal to 13 months.

### Statistical analysis

Childhood leukaemia is a heterogeneous disease defined by various morphological and immunological criteria. The analyses of breastfeeding as well as complimentary feeding characteristics and risk of childhood leukaemia were restricted to ALL since the hypothesis regarding these potential associations is specific for ALL. Given that the peak incidence of ALL as well as its ‘common’ subtype (CD10+, CD19+) is from age 2–5 years (Swensen *et al*, 1997; Smith *et al*, 1999), analyses were also performed utilising only these cases aged 2–5 years at diagnosis and their respective controls. Baseline characteristics of cases and controls were compared with the Pearson chi-square test. To assess the association between breastfeeding and other early feeding patterns and risk of ALL, conditional logistic regression models were used. Odds ratios (ORs) were considered to be consistent with statistical significance if their 95% confidence intervals (CIs) excluded 1.00. Confounding and effect modification were examined by using chi-square tests to compare the likelihood ratio statistics of the models with and without inclusion of confounding factors (annual household income and maternal education) and interaction terms (child's Hispanic status and phase of data collection). *P*-values <0.05 are considered significant for additive models, while *P*-values <0.20 are used for interaction terms in nonadditive models to increase the probability of detecting interactions that may be present. Since child's Hispanic status and phase of data collection were not significant (*P*⩾0.20), an additive conditional logistic model was assumed (Hosmer and Lemeshow, 2000).

## RESULTS

The ALL cases and controls were similar with respect to birth weight and maternal age at birth (Table 1). Compared to controls, more leukaemia cases came from families with lower annual household income (*P*<0.001) and were born to mothers with fewer years of maternal education (*P*=0.07). The study sample consisted of 38% Hispanic, 50% non-Hispanic white, 3% non-Hispanic black, and 9% other. Frequency of ever breastfeeding was 81% in the cases and 84% in the controls.

After adjusting for household income and maternal education, ever compared to never breastfed (OR=0.99; 95% CI=0.64–1.55) and breastfeeding duration in months (OR=1.00; 95% CI=0.98–1.02) were not associated with risk of ALL (Table 2). Similarly, when compared to no breastfeeding, breastfeeding less than or equal to 3 months, 4–6 months, 7–12 months, and greater than 13 months were not associated with ALL risk, and the *P*-value for trend across the categories was not significant. In addition, exclusivity of breastfeeding was examined to assess the independent biological effects of breast milk on the risk of ALL (Table 2). Feeding only breast milk for any length of time was not associated with ALL risk, and no significant trend across the categories existed. Restricting the analysis to ALL cases and their respective controls diagnosed from age 2–5 years revealed elevated, statistically nonsignificant effect estimates for breastfeeding and risk of disease (Table 2). Results for cALL cases and controls (CD10+, CD19+) were also of similar magnitude and direction (not shown).

The associations of complimentary early feeding characteristics with risk of ALL from age 1 to 14 years and age 2–5 years among children who were breastfed were also examined. For both age groups, type of milk/formula used at or before 6 months and after 6 months, age started eating solid foods, and type of solid food had no significant effect on risk of ALL (not shown).

## DISCUSSION

This study investigated the role of breastfeeding and other early childhood feeding patterns as they relate to risk of childhood ALL. Breastfeeding was not associated with risk of ALL among children diagnosed between age 1 and 14 years or between age 2 and 5 years. In children who were breastfed, none of the complimentary feeding characteristics such as type of milk/formula used at or before age 6 months, age started eating solid foods, and type of solid food/baby food was associated with ALL risk.

To date, 13 case–control studies (Davis *et al*, 1988; Magnani *et al*, 1988; van Duijn *et al*, 1988; Shu *et al*, 1995; Petridou *et al*, 1997; Dockerty *et al*, 1999; McKinney *et al*, 1999; Schüz *et al*, 1999; Rosenbaum *et al*, 2000; Hardell and Dreifaldt, 2001; UK Childhood Cancer Study Investigators, 2001; Lancashire and Sorahan, 2003; Jourdan-Da Silva *et al*, 2004) have reported no association between breastfeeding and overall childhood leukaemia or ALL risk. Two of these previous studies restricted their analysis to children diagnosed between age 2 and 5 years and found no significant effect of breastfeeding on risk of leukaemia (Shu *et al*, 1995; UK Childhood Cancer Study Investigators, 2001). In contrast, five case–control studies (Shu *et al*, 1999; Smulevich *et al*, 1999; Infante-Rivard *et al*, 2000; Bener *et al*, 2001; Perrillat *et al*, 2002a, 2002b) have reported a statistically significant protective association between breastfeeding and risk of either childhood ALL or overall leukaemia. One of these studies described a borderline statistically significant effect of breastfeeding on risk of ALL among children diagnosed less than 4 years of age (Infante-Rivard *et al*, 2000). Control selection strategies in all of these breastfeeding studies included the use of population-based (11 studies), hospital-based (five studies), and random digit dial (RDD) (two studies) controls with sample sizes varying from a low of 63 cases of all leukaemia (Davis *et al*, 1988) to a high of 2200 cases of all leukaemia (Shu *et al*, 1999). Most of the statistical analyses adjusted for some measure of socioeconomic status such as household income, social class, parental occupation, or parental education.

In this analysis of NCCLS data, we were able to examine the 2–5 year diagnostic age group, which represents the peak incidence of c-ALL (Swensen *et al*, 1997; Smith *et al*, 1999), and to use detailed exposure assessment of breastfeeding initiation, duration, and exclusivity, the latter of which has not been explored in prior studies. Earlier, we evaluated selection bias by conducting an analysis of 64 pairs of matched birth certificate and friend controls from the study and 192 ‘ideal’ population-based controls chosen randomly from birth records for the study area (Ma *et al*, 2004). After comparing data on parental age, parental education, mother's reproductive history, and birth weight, all variables, except birth weight, showed no significant differences between participating birth certificate and ‘ideal’ controls as compared to that between participating friend and ‘ideal’ controls. These results suggest that the NCCLS birth certificate controls are representative of the source population from which the cases arose.

The prevalence of having ever breastfed in the control group (84%) was greater in the NCCLS than other previous populations studied. For example, the prevalence of breastfeeding in three of the largest studies to date on breastfeeding and risk of childhood leukaemia (United Kingdom Childhood Cancer Study, Children's Cancer Group, and Oxford Survey of Childhood Cancers) was 62, 54, and 49%, respectively (Shu *et al*, 1999; UK Childhood Cancer Study Investigators, 2001; Lancashire and Sorahan, 2003). Descriptive statistics from phase II (1991–1994) of the Third National Health and Nutrition Examination Survey (NHANES III) reported that 54% of US infants were ever breastfed (Li *et al*, 2002). The high prevalence of breastfeeding among the NCCLS controls might have hindered the ability to detect an association of breastfeeding with risk of childhood ALL since most of the controls (and cases) were exposed, that is, breastfed.

An inherent limitation of the NCCLS is that the exposure histories were obtained by self-report and after diagnosis. This is a potential drawback of any case–control study since biologic mothers of cases may recall certain exposures differently (more completely or influenced by the knowledge of their child's diagnosis) than biologic mothers of controls. This bias is unlikely to have been a major concern since there was no public perception at the time of data collection that breastfeeding (Davis, 1998) or other early feeding characteristics might be associated with childhood leukaemia. In addition, we attempted to minimise reporting differences by mailing preparatory materials to serve as an ‘*aide memoire*’ for the respondents prior to the in-home interview.

Strengths of the NCCLS include extremely rapid case ascertainment (within 24–48 h), population-based selection of controls, detailed exposure assessment, and comprehensive analysis of early childhood nutrition in relation to childhood leukaemia risk. We established a hospital-based system of rapid case ascertainment in the source population to shorten the interval from the date of diagnosis to interview to maintain complete (or near complete) case ascertainment in the study area, and to obtain pretreatment biological specimens. Second, the method of selecting controls from the population-based statewide birth registry ensures that controls are identified from the same study base as cases (as detailed above). Third, the study benefited from a detailed exposure assessment of breastfeeding and other early childhood feeding characteristics. The biological mother was asked whether or not she breastfed and how many months she only breastfed, thus allowing the calculation of an exclusive breastfeeding variable for analysis. She was also asked what type of formula or milk her child drank at or before 6 months and after 6 months of age. Finally, we conducted an earlier analysis of childhood dietary data consisting of specific foods/food groups that emphasised the protective benefits of nutrition during the first 2 years of life in relation to risk of childhood leukaemia (Kwan *et al*, 2004a). This current analysis examined breastfeeding, the other important component of a child's early diet, thus providing a complete assessment of the potential associations between postnatal nutritional exposures and childhood leukaemia risk.

In conclusion, this investigation was a comprehensive examination of breastfeeding patterns and the risk of childhood ALL in an essentially population-based case–control study. No association between breastfeeding and risk of ALL was observed. Furthermore, other early childhood feeding characteristics among breastfed children were not associated with ALL risk. Overall, this study did not support the hypothesis that breastfeeding protects against the risk of childhood ALL.

## Figures and Tables

**Table 1:** Selected characteristics of non-infant (age 1–14 years) childhood acute lymphoblastic leukemia (ALL) cases and controls in the Northern California Childhood Leukemia Study (NCCLS), 1995–2002

	**Cases *n* (%)**	**Controls *n* (%)**	***P*-value (*χ*^2^ test)^a^**
*Child age (years)* b			—
1–1.99	26 (8)	34 (8)	
2–5	186 (60)	244 (61)	
6–10	70 (23)	83 (21)	
11–14	29 (9)	39 (10)	
Mean (years)±s.d.	5.5±3.3	5.4±3.3	
			
*Child sex* b			—
Male	164 (53)	208 (52)	
Female	147 (47)	192 (48)	
			
*Child race/ethnicity* b			—
Hispanic	116 (38)	152 (38)	
Non-Hispanic white	157 (50)	200 (50)	
Non-Hispanic black	9 (3)	11 (3)	
Other	29 (9)	37 (9)	
			
*Birth weight (g)*			0.59
<2500	19 (6)	20 (5)	
2500–2999	52 (17)	57 (14)	
3000–3499	107 (36)	140 (36)	
⩾3500	122 (41)	175 (45)	
Unknown	11	8	
Mean (g)±s.d.	3405.8±615.4	3446.1±581.0	
			
*Maternal age at birth (years)*			0.11
<25	108 (35)	110 (28)	
25–34	162 (52)	232 (58)	
≥35	39 (13)	57 (14)	
Unknown	2	1	
Mean (years)±s.d.	28.0±6.0	28.7±5.9	
			
*Maternal education*			0.07
⩽High school	135 (43)	141 (35)	
Some post-high school	89 (29)	137 (34)	
College graduate	86 (28)	122 (31)	
Unknown	1	0	
			
			
			
*Annual household income, $*			<0.001
<15 000	42 (13)	37 (9)	
15 000–29 999	61 (20)	56 (14)	
30 000–44 999	50 (16)	47 (12)	
45 000–59 999	58 (19)	61 (15)	
60 000–74 999	31 (10)	52 (13)	
⩾75 000	69 (22)	147 (37)	
			
Total	311^c^	400^c^	

a Pearson *χ*^2^ test; two-sided.

b For Phase one (19/8/1995–30/11/1999), cases and controls 1 : 1 matched on date of birth, sex, Hispanic ethnic status, maternal race, and maternal county of residence at birth. For Phase two (1/121999–30/11/2002), cases and controls 1 : 1 and 1 : 2 matched on date of birth, sex, Hispanic ethnic status, and maternal race. Age is age at diagnosis for cases and age at the corresponding date for controls.

c A total of 222 1 : 1 case–control pairs and 89 1 : 2 case–control triplets.

**Table 2:** Results of matched analysis for initiation, duration, and exclusive breastfeeding and risk of non-infant childhood acute lymphoblastic leukemia (ALL) from age 1 to 14 years and age 2 to 5 years in the NCCLS, 1995–2002

	**Age 1–14 years**			**Age 2–5 years**		
	**Cases (*n*)**	**Controls (*n*)**	**Adjusted OR (95% CI)^a^**	**Cases (*n*)**	**Controls (*n*)**	**Adjusted OR (95% CI)^a^**
*Breastfeeding initiation*						
Never	57	64	Reference	25	40	Reference
Ever	248	334	0.99 (0.64–1.55)	158	203	1.49 (0.83–2.65)
						
*Breastfeeding duration*						
None	57	64	Reference	25	40	Reference
⩽3 months breastfed	97	115	1.14 (0.68–1.91)	63	73	1.67 (0.85–3.28)
4–6 months breastfed	51	74	0.84 (0.48–1.47)	28	46	1.07 (0.50–2.25)
7–12 months breastfed	56	89	0.88 (0.51–1.53)	34	53	1.29 (0.63–2.67)
⩾13 months breastfed	44	56	1.08 (0.61–1.92)	33	31	1.87 (0.88–3.95)
			*P* trend^b^=0.75			*P* trend^b^=0.34
						
*Breastfeeding duration (in months)*	305	398	1.00 (0.98–1.02)	183	243	1.02 (0.99–1.05)
						
*Exclusive breastfeeding*						
Formula only	57	64	Reference	25	40	Reference
⩽3 months breast milk only	136	180	1.06 (0.65–1.71)	89	110	1.75 (0.91–3.34)
4–6 months breast milk only	49	64	0.97 (0.55–1.71)	32	41	1.32 (0.63–2.77)
7–12 months breast milk only	43	63	0.98 (0.55–1.75)	22	40	1.14 (0.53–2.44)
⩾13 months breast milk only	16	26	0.86 (0.38–1.92)	12	13	2.04 (0.69–6.07)
			*P* trend^b^=0.64			*P* trend^b^=0.74

a OR, odds ratio; CI, confidence interval. OR and CI derived from conditional logistic regression, which accounts for matching on child's date of birth, sex, Hispanic ethnic status, maternal race, and maternal county of residence at birth (only Phase one). Adjusted for annual household income and maternal education.

b Derived from treating the categorical variables on an ordinal scale in the conditional logistic regression model.
